# Integrated network pharmacology and experimental verification to explore the potential mechanism of San Ying decoction for treating triple-negative breast cancer

**DOI:** 10.3724/abbs.2024015

**Published:** 2024-03-21

**Authors:** Xiaojuan Yang, Feifei Li, Youyang Shi, Yuanyuan Wu, Rui Yang, Xiaofei Liu, Yang Zhang, Guangtao Zhang, Mei Ma, Zhanyang Luo, Xianghui Han, Ying Xie, Sheng Liu

**Affiliations:** 1 Institute of Traditional Chinese Medicine Surgery Longhua Hospital Shanghai University of Traditional Chinese Medicine Shanghai 200032 China; 2 Department of Breast Surgery Shanxi Provincial Cancer Hospital Taiyuan 030013 China; 3 Department of Breast Surgery Affiliated Hospital of Shandong University of Traditional Chinese Medicine Jinan 250355 China; 4 Institute of Toxicology School of Public Health Lanzhou University Lanzhou 730000 China

**Keywords:** breast cancer, SYF formula, invasion and metastasis, network pharmacology, molecular docking

## Abstract

Traditional Chinese medicine (TCM) has been used to treat triple-negative breast cancer (TNBC), a breast cancer subtype with poor prognosis. Clinical studies have verified that the Sanyingfang formula (SYF), a TCM prescription, has obvious effects on inhibiting breast cancer recurrence and metastasis, prolonging patient survival, and reducing clinical symptoms. However, its active ingredients and molecular mechanisms are still unclear. In this study, the active ingredients of each herbal medicine composing SYF and their target proteins are obtained from the Traditional Chinese Medicine Systems Pharmacology database. Breast cancer-related genes are obtained from the GeneCards database. Major targets and pathways related to SYF treatment in breast cancer are identified by analyzing the above data. By conducting molecular docking analysis, we find that the active ingredients quercetin and luteolin bind well to the key targets KDR1, PPARG, SOD1, and VCAM1. *In vitro* experiments verify that SYF can reduce the proliferation, migration, and invasion ability of TNBC cells. Using a TNBC xenograft mouse model, we show that SYF could delay tumor growth and effectively inhibit the occurrence of breast cancer lung metastasis *in vivo*. PPARG, SOD1, KDR1, and VCAM1 are all regulated by SYF and may play important roles in SYF-mediated inhibition of TNBC recurrence and metastasis.

## Introduction

According to the latest global cancer data, the number of new breast cancer cases in 2020 reached 2.26 million. Thus, breast cancer has become the world’s most common cancer [1]. Triple-negative breast cancer (TNBC) is a subtype of breast cancer [2] that has a high rate of recurrence and metastasis, a high degree of malignancy, and a poor prognosis [3]. The combination of traditional surgery, radiotherapy, and chemotherapy remains the main treatment for TNBC because of the lack of targeted therapy. However, severe adverse reactions to chemical drugs limit their further development and application [ 4, 5]. Therefore, identifying more effective treatments for TNBC is urgently needed.

According to traditional Chinese medicine (TCM) theory, the main pathogenesis of breast cancer is believed to be “the deficiency of spleen and kidney, and the accumulation of phlegm, blood stasis, and poison”. In general, the TCM “spleen” and “kidney” systems are dampened, as reflected by weakened immunity and reduced nutrient absorption. Consequently, visceral phlegm/inflammation occurs, which results in blood stasis and sporadic accumulation of impairments. The TCM decoction Sanyinfang (SYF) is based on the treatment principle of “invigorating the kidney and spleen, resolving phlegm and detoxification, and promoting blood circulation” and is composed of *Codonopsis pilosula* Nannf., *Atractylodes macrocephala* Koidz., *Poria cocos* Wolf., *Salviae chinensis* Herba., *Curcuma phaeocaulis* Valeton., *Epimedium brevicornu* Maxim., *Solanum nigrum* Linn., *Scutellariae Barbatae* Herba., and *Prunella vulgaris* Linn. Clinical studies have proven that SYF can effectively prolong disease-free survival and reduce the invasion and metastasis of TNBC [6].

Multiple known components of the herbs in SYF have been studied for the treatment of breast cancer. Quercetin is one of the main flavonoids found in *Epimedium brevicornu* Maxim., *Scutellariae Barbatae* Herba., and *Prunella vulgaris* Linn., and it has antioxidant, anti-inflammatory, and anticancer properties [7]. In breast cancer, quercetin can exert antitumor effects by altering cell cycle progression, inhibiting cell proliferation, and inhibiting angiogenesis and epithelial-mesenchymal transition (EMT). Quercetin inhibits the activities of CDK2, cyclin A, and cyclin B, thereby blocking MCF-7 breast cancer cells in S phase and inducing MDA-MB-453 cells to arrest in G2/M phase [ 8, 9]. Quercetin targets the VEGFR-2-mediated angiogenesis pathway, inhibits the expression of the downstream regulator AKT, and inhibits breast cancer growth [ 10, 11]. Moreover, it can inhibit the occurrence of EMT by increasing the expression of E-cadherin and decreasing the expressions of the N-cadherin, vimentin, and snail protein families in many cancers. Moreover, the invasion and migration of tumor cells can be inhibited by regulating the expression of matrix metalloproteinase [ 12, 13].

Luteolin, a natural flavonoid that exists in *Prunella vulgaris* Linn., *Codonopsis pilosula* Nannf., and *Epimedium brevicornu* Maxim., inhibits the growth of breast cancer cells through various mechanisms, such as stimulating cancer cell apoptosis and cell cycle arrest and inhibiting cell invasion and metastasis. Luteolin treatment results in a decrease in mesenchymal markers and an increase in epithelial markers in TNBC cells, inhibiting TNBC cell migration [14]. In addition, luteolin can inhibit the invasion of breast cancer cells by inhibiting VEGF production and the antiangiogenic activity of its receptor [15].

**Figure 1:**
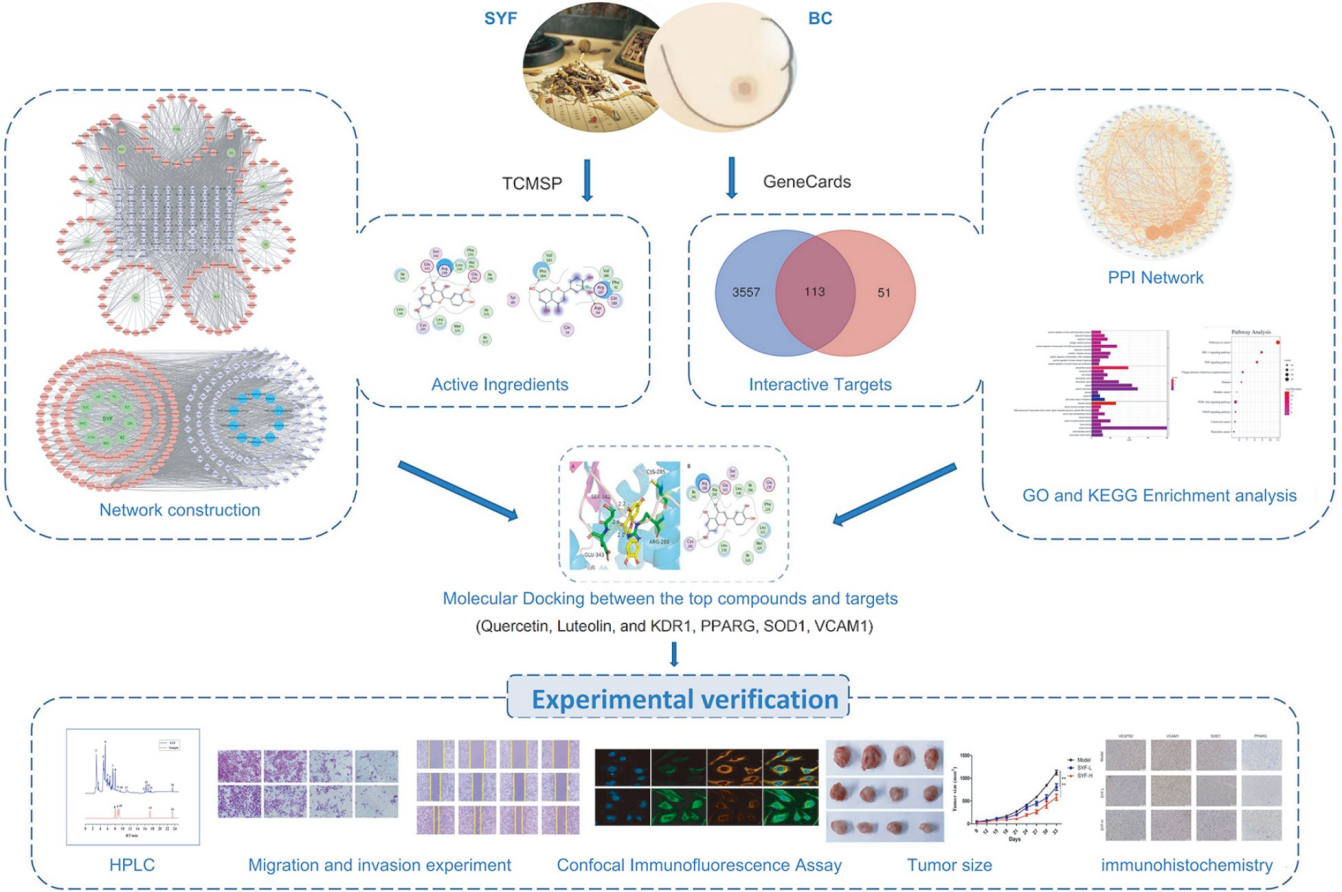
Figure 1 Workflow for dissecting the anti-breast cancer mechanism of SYF

However, the full spectrum of components and their target proteins involved in the mechanism of action of SYF in TNBC treatment has not been investigated. In this study, the active ingredients of SYF and their interactive targets were screened by network pharmacology. Then, molecular docking was used to verify the binding ability of the active ingredients and interactive targets. Finally, *in vitro* and *in vivo* experiments proved that SYF can regulate the expressions of four interactive targets related to invasion and metastasis ( Figure 1).

## Materials and Methods

### Identification of potential active ingredients and related targets of SYF in the treatment of BC

We used the Traditional Chinese Medicine Systems Pharmacology Database (TCMSP) to retrieve the effective chemical ingredients of SYF and their putative action targets. TCMSP is a unique TCM system pharmacology platform that captures the relationships among drugs, targets, and diseases. The TCMSP database includes chemical substances from the herbs and their targeting networks, as well as their oral bioavailability (OB) and drug likeness (DL). OB is the amount of the ingredient that actually enters circulation. The DL model is designed to estimate the potential of a compound to be developed into a drug. The active ingredients of each herb were obtained from the TCMSP database under the following screening conditions: OB≥30% and DL≥0.18. We supplemented the active ingredients by referring to the relevant literature in the CNKI ( https://www.cnki.net/) and PubMed databases. The corresponding target proteins of each ingredient were also obtained from the TCMSP database.

We used “breast cancer” as the keyword to search for BC-related genes in the GeneCards database ( https://www.genecards.org/), which includes detailed information on disease-related genes. Then, we intersected the targets of SYF components and BC-related targets by using Venny 2.1.0 ( http://bioinformatics.psb.ugent.be/webtools/Venn/). The overlapping genes were considered targets of SYF active components in the treatment of BC and were visualized by using a Venn diagram.

### Construction of the “SYF-active ingredients-interactive targets” network

The “SYF-active ingredients-interactive targets” network diagram was constructed for visualization using Cytoscape 3.7.2 based on the interactions among drugs, compounds, and gene symbols. Nodes and edges were generated by the software. The green, pink, and purple nodes represent drugs (SYF and its composing herbs), active ingredients, and gene symbols, respectively. The interactions between SYF and active ingredients and between active ingredients and gene symbols were depicted by edges. Then, we used the network analyzer tool to calculate the degree of each ingredient based on the number of edges between the ingredients and the targets and screened out the top active ingredients. A high degree corresponds to the high importance of the role of the node (ingredient) in the network.

### Construction of the “protein-protein interaction” network

The STRING database ( https://cn.string-db.org/) contains abundant information on known or predicted protein-protein interaction (PPI) relationships. We uploaded the intersecting genes to the STRING website, with the species set as “ *Homo sapiens*,” and obtained the PPI network information. The obtained PPI network data were imported into Cytoscape 3.7.2 for clustering, and core proteins were obtained using the MCODE plug-in.

### Gene Ontology and KEGG pathway enrichment analysis

We uploaded the intersecting genes to the DAVID database ( http://david.ncifcrf.gov/home.jsp) to perform Gene Ontology (GO) and KEGG pathway enrichment analysis. GO analysis revealed three aspects of biology: cellular component, molecular function, and biological process. KEGG ( www.kegg.jp/kegg/kegg1.html) enrichment focuses on biological pathways associated with target genes. The obtained data were visualized by the “SYF-active ingredients-interactive targets-signaling pathways” network in Cytoscape 3.7.2.

### Molecular docking

The active compounds of the medicine are considered small-molecule ligands, and the target proteins act as receptors. Molecular docking is a computational analysis method used to characterize the interaction between a molecule and its protein receptor. We analyzed the docking of two compounds and four targets. The mol2 files of the compounds were downloaded from the PubChem database. The protein targets were imported into the RCSB Protein Data Bank Database ( https://www.rcsb.org) to obtain their 3D structure. Information on the compounds and targets was imported into PyMOL 2.1, and the docking data were analyzed using the AutoDock 1.5.6 plugin. The center position and length, width, and height of the grid box were determined according to the interaction site between the small molecule and the target.

### SYF formula

*Codonopsis pilosula* Nannf. (12 g), *Atractylodes macrocephala* Koidz. (12 g), *Poria cocos* Wolf. (12 g), *Salviae Chinensis* Herba. (30 g), and *Curcuma phaeocaulis* Valeton. (30 g), *Epimedium brevicornu* Maxim. (15 g), *Solanum nigrum* Linn. (30 g), and *Scutellariae Barbatae* Herba. (30 g), and *P. vulgaris* Linn. (9 g) were purchased from Shanghai Wanshicheng Pharmaceutical Co., Ltd. (Shanghai, China) and identified by Wuxi App Tec (Shanghai) Co., Ltd. (Shanghai, China). Morphological, microscopic, and phytochemical identification was performed according to the Pharmacopoeia of the People’s Republic of China (2015 edition). The herbarium was deposited in the TCM Pharmacy Department of Longhua Hospital affiliated with Shanghai University of Traditional Chinese Medicine.

The SYF extract was prepared as follows: the SYF raw medicine materials were weighed, soaked in distilled water (1:10) for 30 min, boiled, simmered at 60°C for 30 min, and subsequently filtered to collect the decoction. The whole procedure was repeated using distilled water (1:8). The two decoctions were combined, concentrated, and freeze-dried to prepare SYF extract powder. The SYF extract powder was reconstituted using pure water before use. Subsequently, the quality of the SYFs was evaluated by HPLC-DAD using a mixed standard sample of icariin, luteolin, quercetin, curcumin, and stigmasterol. The SYF extract contained all of these chemical components ( Supplementary Figure S1).

### Cell culture

The human breast cancer cell line MDA-MB-231 and the murine mammary cancer cell line 4T1 were purchased from the National Collection of Authentic Cell Cultures of the Institute of Biochemistry and Cell Biology, Chinese Academy of Sciences (Shanghai, China). MDA-MB-231 cells were cultured in Dulbecco’s modified Eagle medium (DMEM; Gibco, Carlsbad, USA), and 4T1 cells were maintained in RPMI-1640 (Gibco). The media were supplemented with 10% fetal bovine serum (FBS; Hyclone, Logan, USA), 100 U/mL penicillin, and 100 mg/L streptomycin (Invitrogen, Carlsbad, USA), and the cells were cultured in a humidified incubator at 37°C with 5% CO _2_.

### Cell viability assay

The viability of TNBC cells was assessed by 3-(4,5)-dimethylthiazolyl)-3,5-diphenyltetrazolium bromide (MTT) assay. MDA-MB-231 and 4T1 cells were seeded in 96-well culture plates at a density of 5×10 ^3^ cells/well and incubated for 24 h at 37°C in a 5% CO _2_ atmosphere. The cells were treated with SYF extract (0, 25, 50, 100, 200, or 400 μg/mL) for 24, 48, or 72 h. After treatment, 20 μL of the prepared MTT solution (5 mg/mL) was added to each well incubated at 37°C for another 4 h. The supernatant was removed, and 150 μL of DMSO was added to each well. Cell viability was analyzed by measuring the absorbance at 490 nm with a microplate reader (Synergy H1 Hybrid Reader; BioTek, Winooski, USA). Each experiment was repeated at least three times.

### Wound healing and transwell assays

For migration assays, two breast cancer cell lines were seeded into 6-well plates and cultured until they reached confluence. Horizontal lines were created with 200-μL pipette tips and cell debris was washed away with PBS. Then, the cells were treated with SYF extract (0, 50, 100, or 200 μg/mL) for 24 or 48 h. The extent of cell movement was measured and imaged at 0, 24, and 48 h with an Olympus IX71 microscope (Olympus, Tokyo, Japan).

A transwell assay was used to evaluate the ability of the cells to metastasize and invade. MDA-MB-231 and 4T1 cells were treated with SYF extract (0, 50, 100, or 200 μg/mL) for 24 or 48 h. The upper chambers were coated with 50 μL of Matrigel in the invasion experiment, but no Matrigel was used in the metastasis experiment. Serum-free medium and TNBC cells (5×10 ^4^) were added to the upper chambers. RPMI 1640 or DMEM (500 μL) containing 5% FBS was added to the lower chambers. After 24 h of incubation, the cells were washed twice with PBS, after which the cells in the upper compartment were removed with a cotton swab, and the migrated or invaded cells were fixed with 4% paraformaldehyde and stained with crystal violet. Finally, the number of migrated or invaded cells were counted under a microscope (Olympus).

### Immunofluorescence staining

Log-phase 4T1 and MDA-MB-231 cells were harvested and resuspended. A total of 200 μL of suspended cells was then pipetted into 35-mm chamber slides at a density of 1×10 ^5^ cells/mL. After being treated with DMEM or SYF (100 μg/mL) for 24 h, the cells were fixed with 4% paraformaldehyde for 30 min at room temperature. The cells were permeabilized with 0.5% Triton X-100 for 5 min and blocked with BSA for 1 h at room temperature. Between each step described above, the cells were washed 3 times (5 min each) with PBS. F-actin was stained with Alexa Fluor™ 488 Phalloidin for 1 h for visualization. The cells were then counterstained with 4 mg/mL 4,6-diamidino-2-phenylindole (DAPI).

After treatment, 4T1 and MDA-MB-231 cells were fixed in 4% paraformaldehyde for 5 min, permeabilized with 0.5% Triton X-100 for 5 min, and blocked with BSA for 1 h at room temperature. The cells were then incubated with primary antibodies against E-cadherin, vimentin (1:50 dilution; Proteintech, Chicago, USA) overnight at 4°C, followed by incubation with fluorescence-labeled secondary antibody for 1 h. After washing, the samples were stained with DAPI and images were captured using a confocal microscope (Zesis LSM-800, Oberkochen, Germany).

### Animal experiment

Female BALB/c mice (6–8 weeks old) were obtained from the Shanghai Laboratory Animal Center (Shanghai, China) and maintained on cycles of 12 h of light (7 am to 7 pm) and 12 h of darkness (7 pm to 7 am). Animal experiments were conducted in accordance with the animal certification procedures of the Animal Welfare Ethics Committee of Shanghai University of Traditional Chinese Medicine (permit No. PZSHUTCM200717028). The mice were randomly divided into four groups according to body weight, with four mice in each group. Except those in the normal group, the right fat pad of number 2 mammary gland of mice were injected with 2×10 ^5^ 4T1 cells suspended in 100 μL of PBS to establish a xenograft mouse model. The normal group (without injection of tumor cells) and the model group (with injection of tumor cells) were given normal saline orally every day, and the low- and high-dose SYF groups were given 2.7 and 10.8 g/kg/d herbal extract per oral gavage, respectively. Tumor volume (V=0.5×L×W ^2^, where L and W are the tumor length and width, respectively) and body weight were measured every three days. All xenograft animals were treated for a period of 5 weeks. At the end of the experiment, all mice were euthanized by cervical dislocation. The tumor tissues of mice in each group were removed, photographed, weighed, and then fixed with 4% paraformaldehyde for further analysis. The main organs (lung, liver, kidney, heart, and spleen) were fixed with 4% paraformaldehyde, after which pathological staining was performed.

### Immunohistochemical staining

The paraffin-embedded tissues were sectioned at 4 mm thickness and arranged on glass slides in sequence. The slides were incubated at 60°C for 6 h, deparaffinized with xylene, and rehydrated in graded ethanol and 3% hydrogen peroxide to block endogenous peroxidase activity. The sections were submerged in citrate or EDTA buffer and microwaved for antigen retrieval. Goat serum (ZSGB-BIO, Beijing, China) was used to block nonspecific binding, and the sections were subsequently incubated at 4°C with specific primary antibodies against KDR1 (1:200; Proteintech), PPARG (1:200; Proteintech), SOD1 (1:200; Proteintech), or VCAM1 (1:200; Proteintech). After incubation with a 1:200 dilution of HRP-conjugated secondary antibodies (Proteintech) was performed. Immediately thereafter, 3,3-diaminobenzidine substrate (DAB; ZSGB-BIO) was achieved for color development, and a counterstain with Mayer’s hematoxylin was performed. All the slides were assessed by two urological pathologists.

### Western blot analysis

After tumor tissue or 4T1 or MDA-MB-231 cells were treated with SYF (0, 50, 100, or 200 μg/mL) for 48 h, total protein was extracted from tissues or cells using lysis buffer containing protease inhibitor cocktail (Beyotime, Shanghai, China). The protein concentration was determined using a BCA Analysis kit (Beyotime), and the protein lysate (20 μg) was subject to western blot analysis with standard protocols. The primary antibodies used for analysis were antibodies against KDR1 (1:1000; Proteintech), PPARG (1:1000; Proteintech), and SOD1 (1:1000; Proteintech). The secondary antibody was goat anti-rabbit secondary antibody conjugated to horseradish peroxidase (1:5000; Proteintech). The protein bands were detected using enhanced chemiluminescence (ECL) reagent (ABclonal, Wuhan, China) on a Bio-Rad ChemiDoc Touch Imaging System (Hercules, USA).

### Statistical analysis

Data analyses were performed with SPSS 24.0 software (SPSS, Chicago, USA) and GraphPad Prism 5.0 (GraphPad Software, San Diego, USA). The differences between groups were determined by one-way ANOVA or Student’s *t* test. Data are presented as the mean±SD. A *P* value less than 0.05 was considered statistically significant.

## Results

### Potential active ingredients and targets of SYF in the treatment of BC

From the TCMSP database, we collected the active ingredients of each drug in SYF with an OB≥30% and a DL≥0.18 as the screening criteria. We identified 21 active ingredients of *Codonopsis pilosula* Nanf., 15 of *Poria cocos* Wolf., 23 of *Epimedium brevicornu* Maxim., 29 of *Scutellariae Barbatae* Herba., 11 of *Prunella vulgaris* Linn., 7 of *Solanum nigrum* Linn., 7 of *Atractylodes macrocephala* Koidz., and 16 of *Salviae Chinensis* Herba. Only 3 components of *Curcumae Rhizoma* were identified under these conditions. Therefore, we supplemented the diet with 21 components reported in previous literature. In total, 135 active ingredients of SYF were obtained for further studies ( Supplementary Table S1). The corresponding target proteins of the active ingredients were obtained from the TCMSP database, and the gene names of the target proteins were uniformly transformed through the UniProt database. Sixteen ingredients without target information were excluded. Repeated targets of different ingredients were combined. A total of 164 gene targets of SYF were ultimately obtained ( Supplementary Table S2).

**Figure 2:**
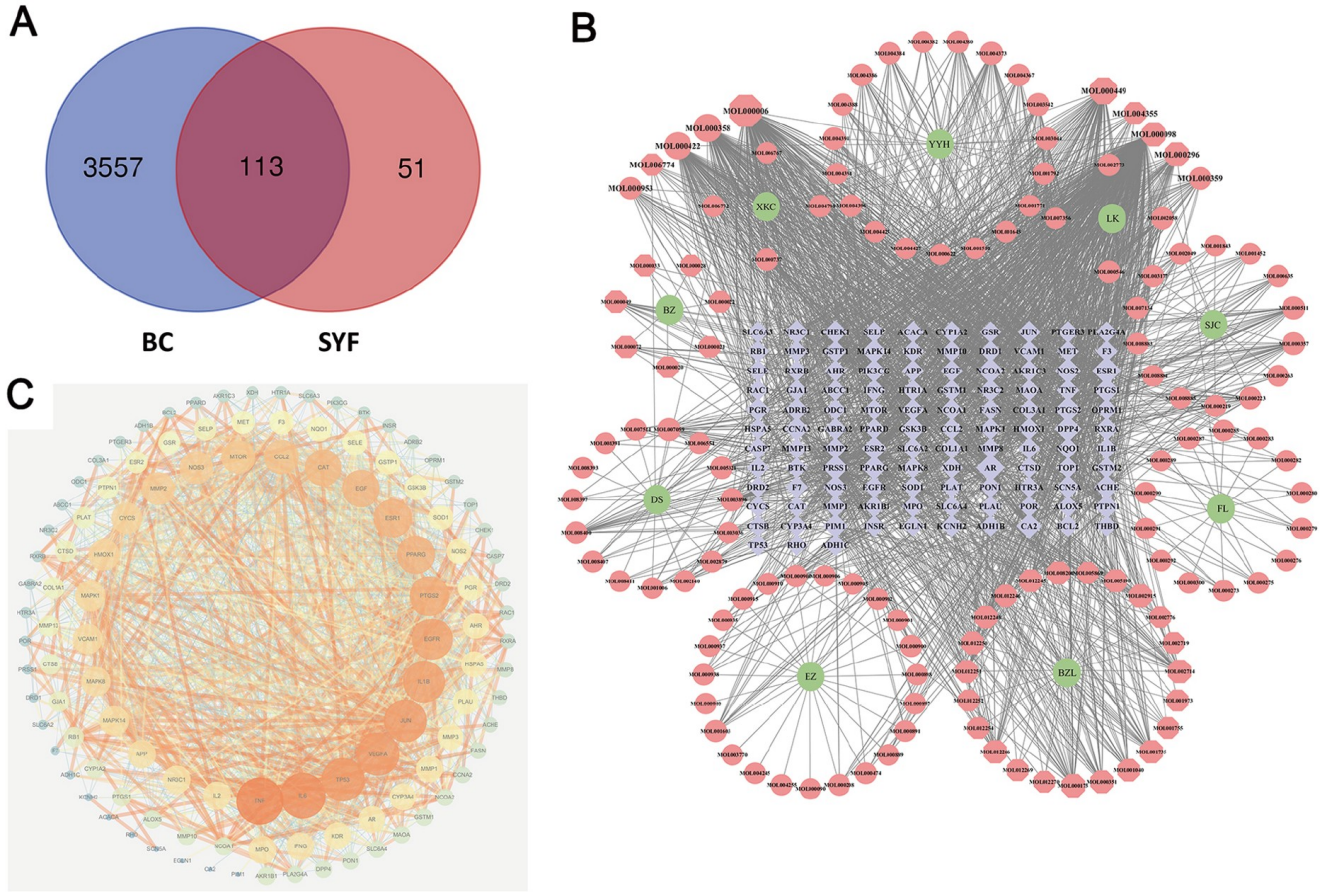
Figure 2 Network pharmacological research on the anti-breast cancer target SYF (A) Venn diagram of drug-disease interaction targets. (B) Network diagram of “SYF-active ingredients-interactive targets”. Pink nodes represent SYF and its active ingredients, and 113 purple nodes represent the overlapping genes between BC and SYF, with gray edges indicating that nodes can interact. (C) The PPI network was constructed according to the degree value. A deeper orange color and larger nodes indicate stronger correlations, and these may be critical genes for the SYF treatment of BC.

With the use of “breast cancer” as the search term, a total of 3670 BC-related genes were obtained from the GeneCards database ( Supplementary Table S3). As shown in the Venn diagram, we contrasted the obtained 164 drug targets with 3670 BC-related genes to obtain 113 overlapping genes, which are likely the most critical genes for SYF-related treatment of BC ( Figure 2A and Supplementary Table S4).

### “SYF-active ingredients-interactive targets” network

To demonstrate how SYF counteracts BC, we constructed a “SYF-active ingredient-interactive target” network using Cytoscape 3.7.2 ( Figure 2B). The top active ingredients were screened based on their degree, which represents the number of related targets of each ingredient. The top active ingredients are quercetin, luteolin, beta-sitosterol, kaempferol, stigmasterol, hederagenin, dinatin, 7-methoxy-2-methyl isoflavone, anhydroicaritin, and baicalein ( Supplementary Table S5).

### Protein-protein interaction network

The PPI network of the 113 overlapping genes was constructed with the STRING database. These genes may be critical targets for SYF-based treatment of breast cancer. The original action relationship map obtained from the STRING database included 113 nodes and 1324 edges ( Supplementary Figure S2). The degree indicates the number of other genes related to one gene, suggesting its importance for treatment. The mean value of the 113 overlapping genes was 23.4, and the average local clustering coefficient was 0.63. The interactions between the proteins were revealed by constructing a network and analyzing it using Cytoscape 3.7.2, arranged according to degree values ( Figure 2C). The core 25 genes with higher degree values are shown in the inner loop. The genes with a deeper red color and larger nodes may be more important in the SYF treatment of breast cancer. Detailed information about the genes and topological indices is provided in Supplementary Table S6.

### GO and KEGG pathway enrichment analysis

**Figure 3:**
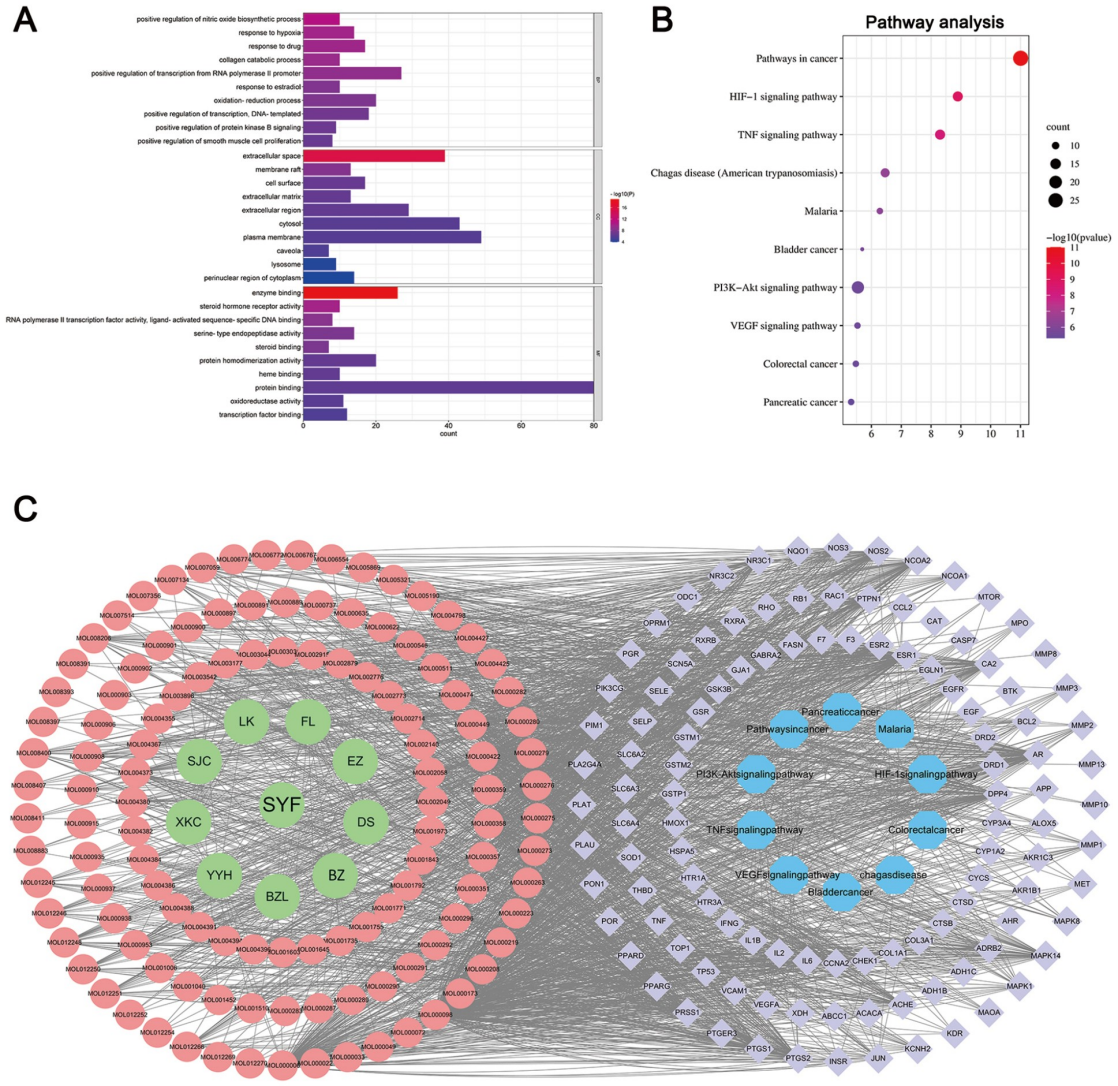
Figure 3 GO and KEGG pathway enrichment analysis (A) Bar graph of the top 10 GO functional enrichment results for the overlapping targets. The X-axis indicates the number of genes enriched in the pathway. The Y-axis represents GO terms. (B) Bubble graph of the KEGG enrichment of overlapping targets. The X-axis is the number of genes enriched in the pathway. The Y-axis represents KEGG pathways. (C) “SYF-active ingredient-interactive target-signaling pathway” network. The green nodes represent SYF and its composition, and the red nodes represent the active ingredients of SYF. The 113 purple nodes represent the overlapping genetic symbols between BC and SYF. The blue nodes represent overlapping gene enrichment signaling pathways.

The 113 overlapping genes were imported to the DAVID database for GO and KEGG pathway enrichment analysis at *P*<0.05. The GO enrichment analysis included cellular component, molecular function, and biological process. A total of 281 biological process (BP), 50 cell component (CC), and 75 molecular function (MF) terms were enriched. The 10 pathways most significantly enriched in each category are presented in Figure 3A. These pathways included the following: BP, positive regulation of transcription from the RNA polymerase II promoter; oxidation-reduction process; signal transduction; positive regulation of transcription; DNA template; and response to drug; CC, plasma membrane; nucleus; cytoplasm; cytosol; and extracellular space; and MF, protein binding, enzyme binding, zinc ion binding, protein homodimerization activity, and identical protein binding. The detailed results are provided in Supplementary Table S7.

KEGG pathway analysis revealed that 93 pathways are involved in SYF treatment of BC ( *P*<0.05), and the 10 pathways related to the genes exhibiting the most significant enrichment are shown in Figure 3B. These pathways included pathways involved in cancer, the HIF-1 signaling pathway, and the TNF signaling pathway ( Figure 3B). The details of all the KEGG pathway enrichment analyses are provided in Supplementary Table S8. By using the 10 pathways related to the genes exhibiting the most significant enrichment, we constructed the “SYF–active ingredients–interactive targets–signaling pathways” network by using Cytoscape 3.7.2 ( Figure 3C).

### Molecular docking analysis

**Figure 4:**
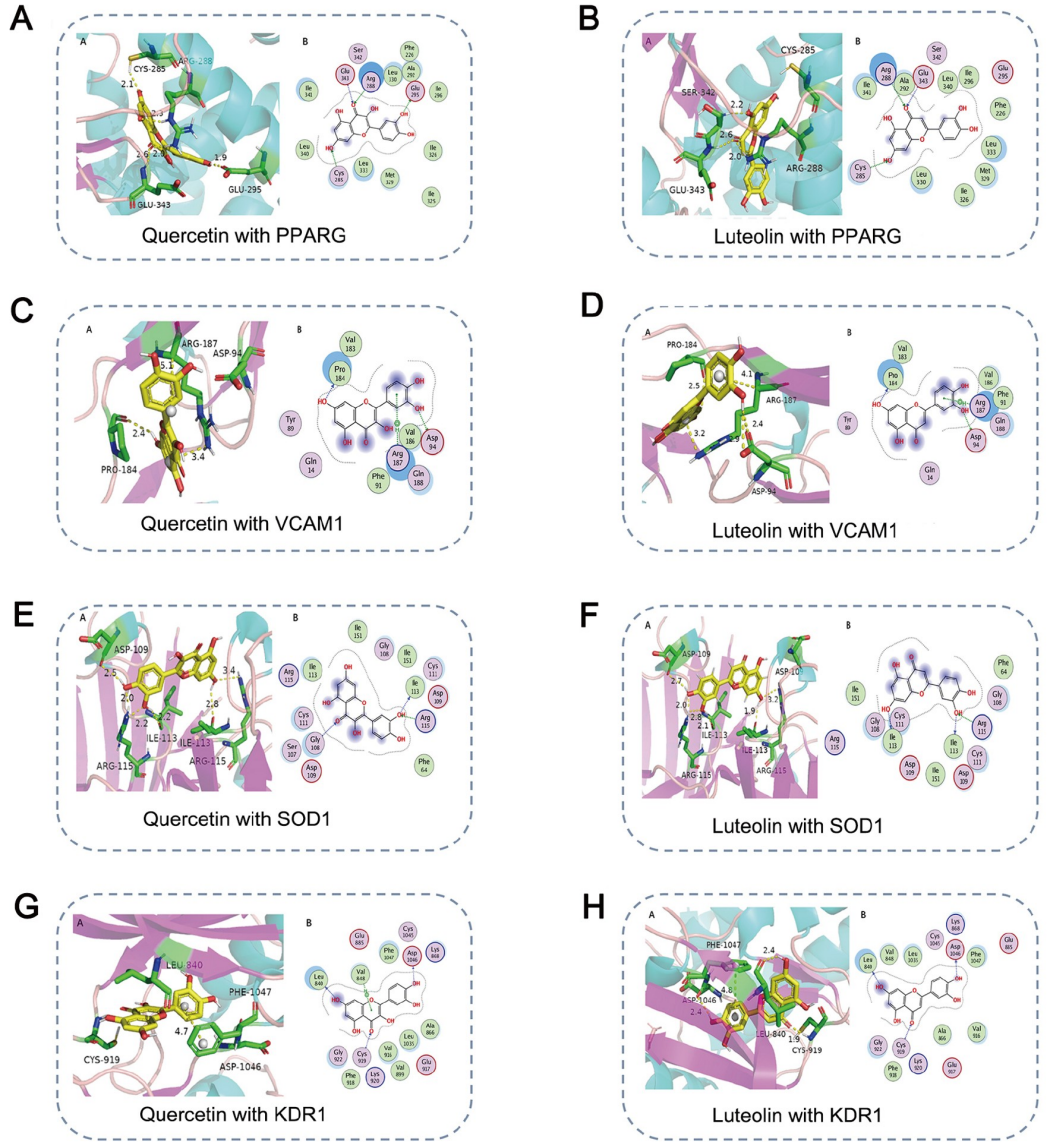
Figure 4 3D and 2D molecular docking binding modes (A) Quercetin and PPARG. (B) Luteolin and PPARG. (C) Quercetin and VCAM1. (D) Luteolin and VCAM1 levels. (E) Quercetin and SOD1. (F) Luteolin and SOD1. (G) Quercetin and KDR1. (H) Luteolin and KDR1.

Using molecular docking analysis, we selected the two active ingredients with the highest degrees, *i.e*., quercetin and luteolin, to study their binding to targets related to invasion, metastasis, and angiogenesis. These proteins included vascular endothelial growth factor (VGFR2/KDR), peroxisome proliferator-activated receptor gamma (PPARG), superoxide dismutase 1 (SOD1), and vascular cell adhesion protein 1 (VCAM1). Quercetin and luteolin are similar in structure, with only one hydroxyl group. The results of molecular docking showed that both active ingredients strongly bind to each target, and the binding energy was less than −6 kcal/mol ( Supplementary Table S3). The binding modes between major reactive chemical groups and their interacting amino acid residues were visualized using PyMOL 2.1 ( Figure 4).

### SYF suppressed the viability, migration, and invasion of TNBC cells

**Figure 5:**
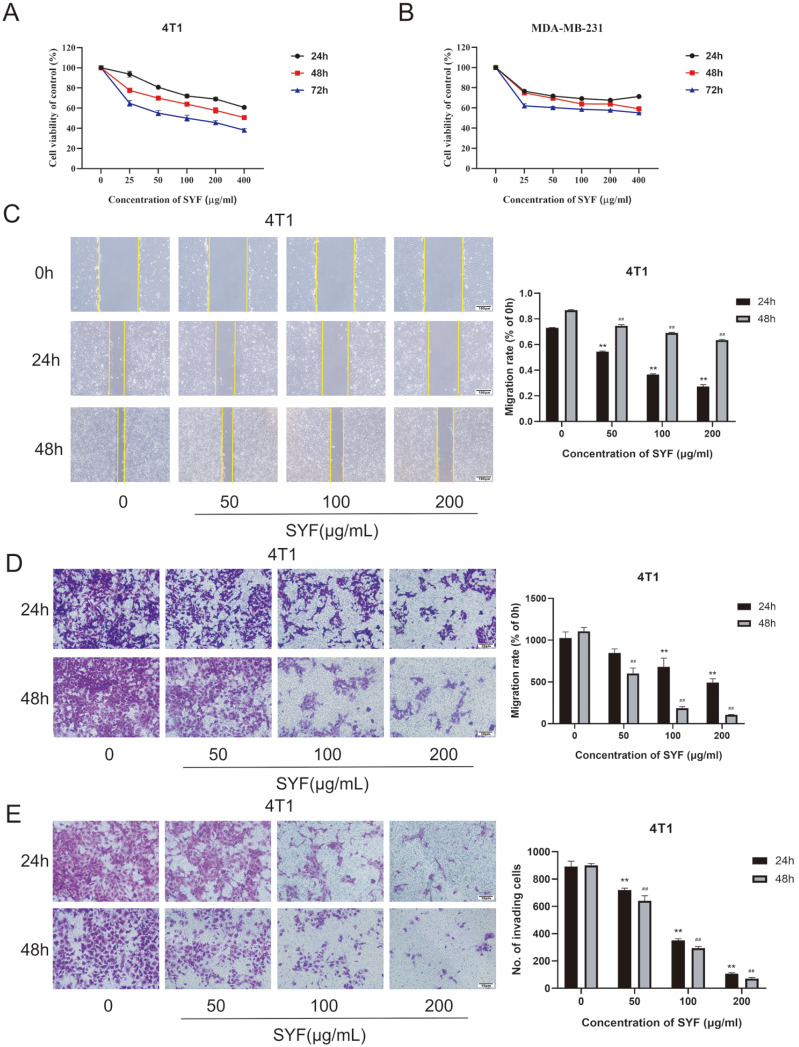
Figure 5 Effects of SYF extract on the proliferation, migration, and invasion of 4T1 and MDA-MB-231 breast cancer cells (A,B) After 24, 48, and 72 h of treatment with SYF extract, the viability of 4T1 and MDA-MB-231 cells was detected by MTT assay. (C,D) The migration of 4T1 cells treated with SYF was analyzed by wound healing assay (100×) and transwell assay (200×). (E) Invasion of 4T1 cells treated with SYF was analyzed by invasion assay using a Matrigel-coated transwell insert (200×). **P<0.01 vs the control group after 24 h; ##P<0.01 vs the control group after 48 h.

We first used MTT assay to assess the effect of SYF extract on the viability of TNBC cells *in vitro*. SYF extract significantly reduced the viability of two TNBC cell lines, mouse 4T1 cells and human MDA-MB-231 cells, in a time- and dose-dependent manner ( Figure 5A,B).

Subsequently, the effect of SYF extract on the metastatic capacity of TNBC cells was further investigated. Through a wound healing assay, we demonstrated that SYF extract significantly reduced the migration ability of MDA-MB-231 and 4T1 cells ( Figure 5C and Supplementary Figure S3A). Migrating ability can also be determined using transwells without Matrigel. Cells seeded in the upper chamber migrated through the holes when attracted by nutrients in the lower chamber. The SYF extract similarly inhibited the migration of both cell lines ( Figure 5D and Supplementary Figure S3B). Moreover, by using the same transwells but coated with Matrigel, we analyzed the invasiveness of the cells. The results revealed that the invasion of both cell lines was significantly decreased by SYF extract ( Figure 5E and Supplementary Figure S3C). The inhibitory effects on the migration and invasion of both cell lines were also time- and dose dependent. These data indicated that SYF extract significantly inhibited the metastatic potential of TNBC cells *in vitro*.

### SYF extract inhibits EMT in breast cancer cells

**Figure 6:**
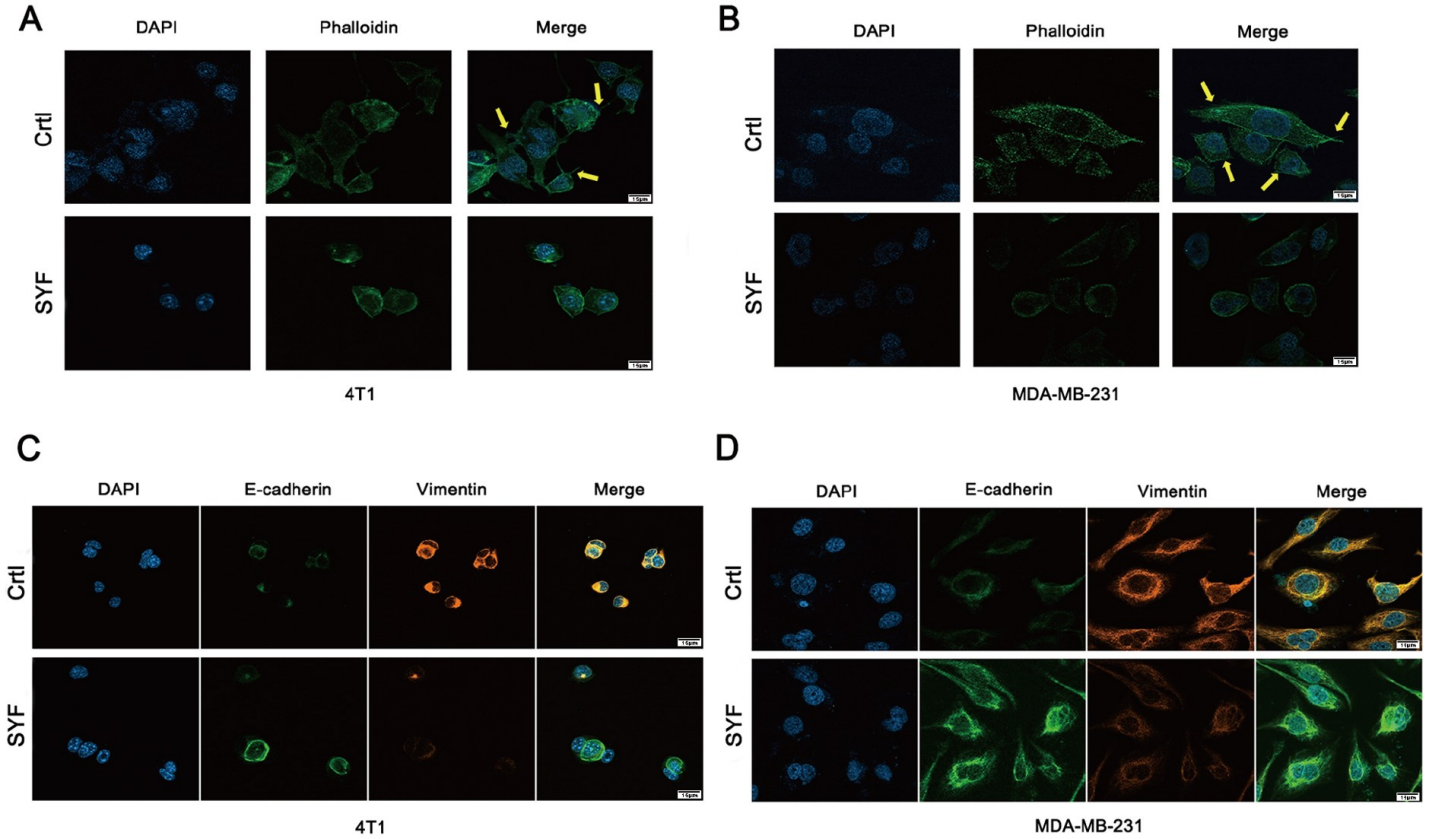
Figure 6 SYF extract inhibits the occurrence and development of EMT (A,B) SYF impairs F-actin cytoskeleton organization in 4T1 and MDA-MB-231 cells (630×). (C,D) Immunofluorescence staining experiments indicated that SYF increased the expression of E-cadherin and decreased the expression of vimentin. E-cadherin (green fluorescence) and vimentin (orange fluorescence) are located mainly in the cytoplasm, and DAPI (blue fluorescence) is located in the nucleus (630×).

The EMT is a biological process closely related to tumor invasion and metastasis. Actin cytoskeleton rearrangement, which can significantly enhance cell motility, plays an important role in the EMT process. Therefore, we investigated the effects of SYF on F-actin microfilaments in 4T1 and MDA-MB-231 cells. The control cells exhibited regular aggregation of F-actin ( Figure 6A,B). When the cells were treated with 100 μg/mL SYF, F-actin fiber expression and lamellipodium formation were significantly reduced.

Immunofluorescence experiments indicated that, compared with that in the control group, the E-cadherin protein expression in the SYF group was significantly upregulated, whereas the vimentin expression was significantly downregulated ( Figure 6C,D). These results indicated that SYF may significantly inhibit the occurrence and development of EMT.

### SYF extract can suppress the growth of breast cancer *in vivo*

**Figure 7:**
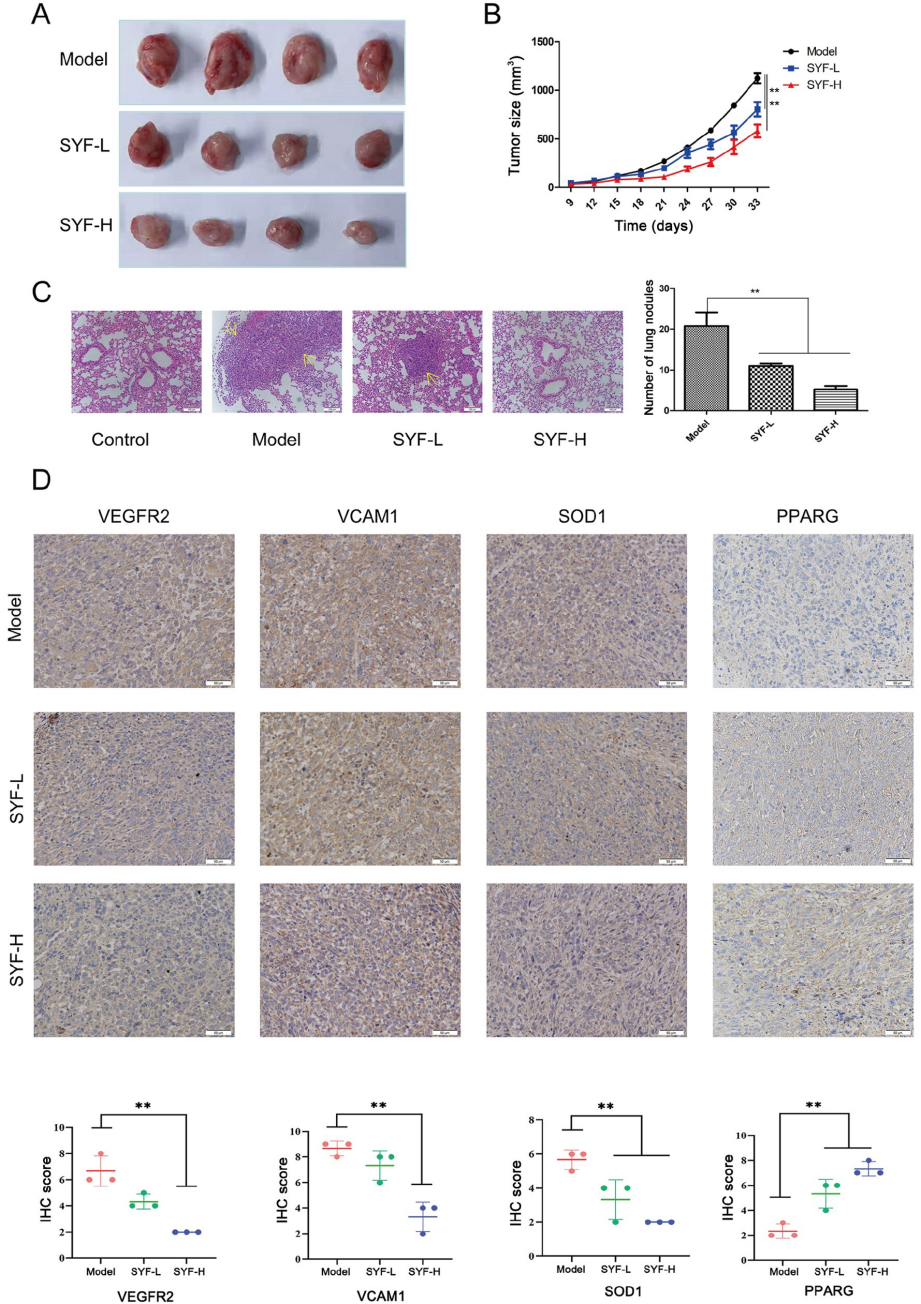
Figure 7 SYF treatment suppresses 4T1 tumor growth and lung metastasis *in vivo* (A) Images of tumors from the different treatment groups after the mice were sacrificed. (B) Tumor size was measured every 3 days from day 9 to day 33 after implantation of 4T1 cells and calculated as length×width×width/2. (C) H&E staining images of lung metastatic tissues and the number of lung metastatic nodules in mice in different treatment groups (20×). (D) Immunohistochemical staining of PPARG, VCAM1, KDR1, and SOD1 in tumor sections from the different treatment groups (40×). **P<0.01.

The antitumor effects of SYF extract were further investigated *in vivo* using a xenograft tumor model of 4T1 cells. The tumor growth rate was significantly reduced after treatment with SYF extract at 2.7 and 10.8 g/kg/day compared with that in the model group ( Figure 7A,B). At the end of the experiment, we measured the volume and weight of tumors isolated from each group of mice. Compared with those in the model group, the volume and weight of the tumors in the SYF extract treatment group were significantly lower ( Supplementary Figure S4A,B), while the body weight of the mice in each group did not change significantly ( Supplementary Figure S4C). Using immunohistochemical (IHC) staining, we further analyzed the expression levels of VEGFR2, PPARG, SOD1, and VCAM1 in 4T1 mouse tumors after SYF treatment. Compared with those in the model group ( Figure 7D), the expression levels of VEGFR2 and VCAM1 in breast cancer tissues were significantly decreased after SYF-H treatment. Moreover, SOD1 was downregulated and PPARG was upregulated after both SYF-L and SYF-H interventions.

Moreover, H&E staining of lung metastases revealed that SYF extract could significantly reduce the number of pulmonary nodules, indicating that SYF extract can inhibit lung metastasis in patients with breast cancer ( Figure 7C) and that SYF-H treatment can reduce the lung indices ( Supplementary Figure S4D). Additionally, no pathological changes occurred in the main organs of the mice in different treatment groups, which demonstrated that SYF extract is not toxic at the organ level ( Supplementary Figure S4E).

### SFY regulates the expressions of VEGFR2, PPARγ, and SOD1 in TNBC cells and tumors

**Figure 8:**
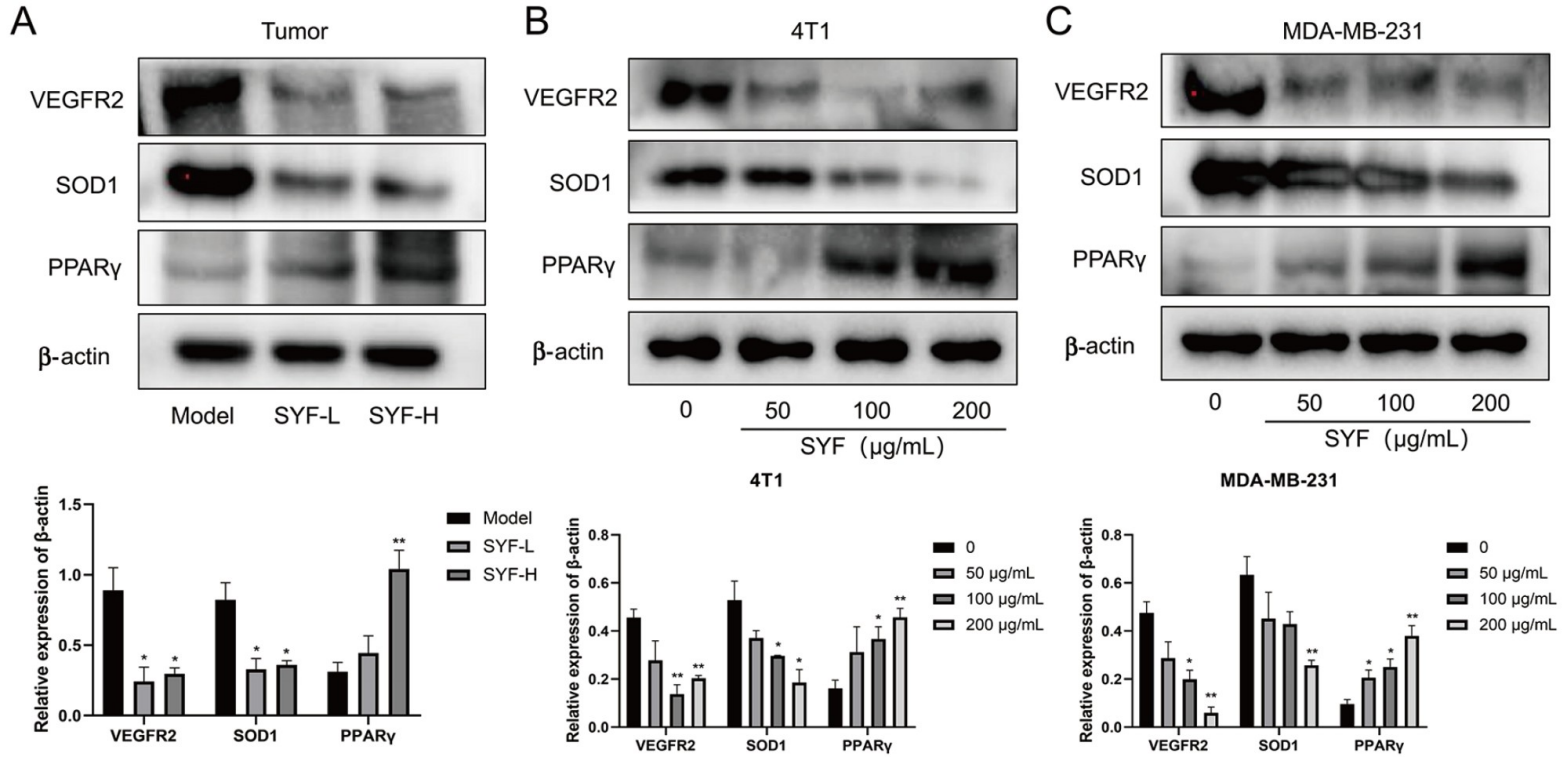
Figure 8 SFY regulates the expressions of VEGFR2, PPARγ, and SOD1 in TNBC cells and tumors (A–C) Western blot analysis was used to evaluate the effect of SYF on the expressions of VEGFR2, PPARγ, and SOD1 in TNBC tumors and cells.

We analyzed the effect of SYF treatment on the protein expressions of VEGFR2, PPARγ, and SOD1 in TNBC cells and tumor tissues. Western blot analysis results showed that SYF treatment significantly reduced the expressions of VEGFR2 and SOD1 in tumors compared with that in the model group. SYF-H increased the expression of PPARγ protein in tumors ( Figure 8A). We treated MDA-MB-231 and 4T1 cells with SYF at concentrations of 50, 100, and 200 μg/mL for 48 h *in vitro*. The results showed that the expression level of PPARγ protein was significantly upregulated with increasing concentrations of SYF, and the expressions of VEGFR2 and SOD1 were downregulated ( Figure 8B,C). These results showed that SYF may inhibit the invasion and metastasis of breast cancer cells by regulating the expressions of VEGFR2, PPARγ, and SOD1.

## Discussion

Network pharmacology can be used to efficiently screen the effective ingredients of TCMs, thus enabling us to explore the potential targets and biological effects of the active ingredients [16]. Molecular docking technology simulates the geometric matching and energy matching of small-molecule ligands and protein receptors through stoichiometric methods to recognize intermolecular interactions, thereby predicting the structure of receptor-ligand complexes [17]. Our study used network pharmacology and molecular docking to explore the active ingredients, targets, and signaling pathways of SYF, thus providing a basis for exploring the molecular mechanism of its effect on TNBC.

We found that the molecular mechanism of SYF in preventing TNBC involves an interaction network with multiple components, pathways, and targets. On the basis of the degree in the “SYF-active ingredients-TNBC related targets” network, the key active ingredients in SYF were quercetin, luteolin, beta-sitosterol, kaempferol, stigmasterol, hederagenin, and dinatin, with quercetin and luteolin ranking the highest. Considering the importance of these two compounds, quercetin and luteolin were identified in our SYF based on reliable compound standards (purity ≥99%). On the basis of the PPI network, most of the proteins, including KDR1, PPARG, SOD1, and VCAM1, are related to angiogenesis, invasion and migration. Molecular docking studies revealed that the key active ingredients (quercetin and luteolin) can bind stably with targets (KDR1, PPARG, SOD1, and VCAM1).

Angiogenesis is one of the main signs of cancer. It promotes the proliferation, invasion, and migration of cancer cells. Tumor angiogenesis is regulated by the fine-tuned balance between proangiogenic and antiangiogenic factors produced by host and tumor cells, including the kinase insertion domain receptor (KDR1) [18]. Under normal physiological conditions, KDR and KDR1 in the body are relatively balanced, and blood vessels are generally not long. However, in tumor tissues, due to changes in the microenvironment, a large amount of KDR is expressed in tumor cells, which activates the “angiogenesis switch”, and KDR1 is directly involved in the formation of tumor blood vessels in the vascular endothelium [ 19, 20]. Breast cancer epithelial cells and stromal cells produce VEGF and express the VEGF receptors Flt-1 and KDR, indicating that VEGF can play a role not only in angiogenesis but also as an autocrine/paracrine regulator of breast cancer, thereby promoting tumor proliferation and invasion [21].

Vascular cell adhesion molecule 1 (VCAM-1) is involved in tumor angiogenesis. It is widely expressed in endothelial and mesenchymal cells and induces EMT and transendothelial migration in cancer [22]. Studies have shown that overexpression of VCAM1 can enhance tumor invasion and migration and promote tumor metastasis [22]. Suppressing the expression of VCAM1 can reduce the proliferation ability of tumors [23]. The percentage of patients with positive VCAM1 expression in breast cancer tissues is increased, and the expression rate is greater in patients with lymph node metastasis. VCAM1 may be related to poor prognosis and tumor metastasis [24].

Peroxisome proliferator-activated receptor gamma (PPARG) is the peroxisome proliferator-activated receptor gamma (peroxisome proliferator)-activated receptor γ (PPARγ) gene. PPARγ is a ligand-dependent nuclear transcription factor that is a member of the nuclear hormone receptor superfamily and has strong anti-inflammatory and antiangiogenic effects [25]. Studies have shown that PPARG is expressed at low levels in a variety of tumor tissues, and PPARγ can inhibit tumor angiogenesis, inhibit tumor cell proliferation, and promote apoptosis after being activated by ligands [26]. In ER-resistant breast cancer, anti-PPARG therapy can promote cell apoptosis [27]. VSP-17, an agonist of PPARγ, inhibits the migration and invasion of TNBC cells by inhibiting the EMT process [28]. In patients with breast cancer, PPARγ expression has a significant beneficial effect on recurrence-free survival [29]. Similarly, PPARγ level is lower in patients with local recurrence than in those who are disease free. PPARγ expression is inversely associated with histological grade in invasive breast carcinoma [30]. Thus, PPARG is a potential therapeutic target for breast cancer treatment.

Previous studies have reported that SOD1 is highly expressed in different types of malignant tumors and can effectively remove superoxide anion free radicals in cells and promote the growth of cancer cells [31]. Recent studies showed that breast cancer cell-derived survivin upregulates the expression of SOD1 in fibroblasts and subsequently transforms it into myofibroblasts, which in turn induces breast cancer progression *in vitro* and *in vivo* [32]. Therefore, targeting SOD1 may be a potential breast cancer treatment strategy.

The above PPARG, VCAM1, KDR1, and SOD1 proteins can affect the proliferation, migration, and invasion of cancer through different mechanisms and are related to the prognosis and metastasis of patients. Therefore, follow-up experimental verification was performed. Our research revealed that SYF can reduce the *in vitro* proliferation of two TNBC cell lines while inhibiting their migration and invasion capabilities. Immunofluorescence experiments showed that SYF significantly increased the protein expression level of E-cadherin and decreased the protein expression of vimentin. We believe that SYF may inhibit the migration and invasion of TNBC cells by inhibiting the EMT process. The expressions of PPARG, VCAM1, KDR1, and SOD1 were detected in the tumor tissues of TNBC mice, and the results showed that SYF can significantly upregulate the expression of PPARG while downregulating the expression levels of VCAM1, KDR1, and SOD1, which is consistent with the network pharmacology and molecular docking results.

Nevertheless, our research has some limitations. First, the number of animals used in this study was too small and should be increased in the future. Second, our study was only an initial exploratory study and lacked validation of specific active compounds. Additional experiments are needed to explore the relationship between these compounds and TNBC and its specific mechanism of action.

In summary, we combined network pharmacology, molecular docking, and *in vivo* and *in vitro* experimental verification to efficiently and accurately explore the potential molecular mechanism of SYF in the treatment of breast cancer. The results show that SYF can reduce the proliferation, migration, and invasion of TNBC cells, delay tumor growth, and inhibit the lung metastasis of breast cancer cells. PPARG, SOD1, KDR1, and VCAM1 are regulated by SYF and may play important roles in SYF-mediated inhibition of TNBC recurrence and metastasis. This research provides a basis for the research and development of new anticancer drugs.

## Supporting information

23489Supplementary_Table_S3

23489Supplementary_Table_S9

23489Supplementary_Table_S8

23489Supplementary_Table_S6

23489Supplementary_Table_S1

23489Supplementary_Table_S7

23489Supplementary_figures

23489Supplementary_Table_S2

23489Supplementary_Table_S5

23489Supplementary_Table_S4

## Supplementary Data

Supplementary data is available at *Acta Biochimica et Biophysica Sinica* online.
