# Supplementary material for: Integrated network pharmacology and experimental verification to explore the potential mechanism of San Ying decoction for treating triple-negative breast cancer: San Ying decoction for treating triple-negative breast cancer
Source: Acta Biochim Biophys Sin (Shanghai). 2024 Mar 21;56(5):763–75. doi: 10.3724/abbs.2024015 (PMC11177106; doi:10.3724/abbs.2024015)
Supplement: 23489Supplementary_figures [file 23489Supplementary_figures.pdf]

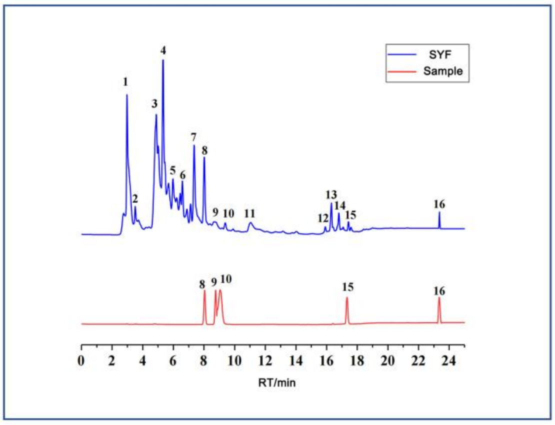

Supplementary Figure S1. **HPLC chromatograms of STF and mixed standard sample at 260 nm. The mixed standard sample consisted of icariin (8), luteolin (9), quercetin (10), curcumin (15), and stigmasterol (16)**

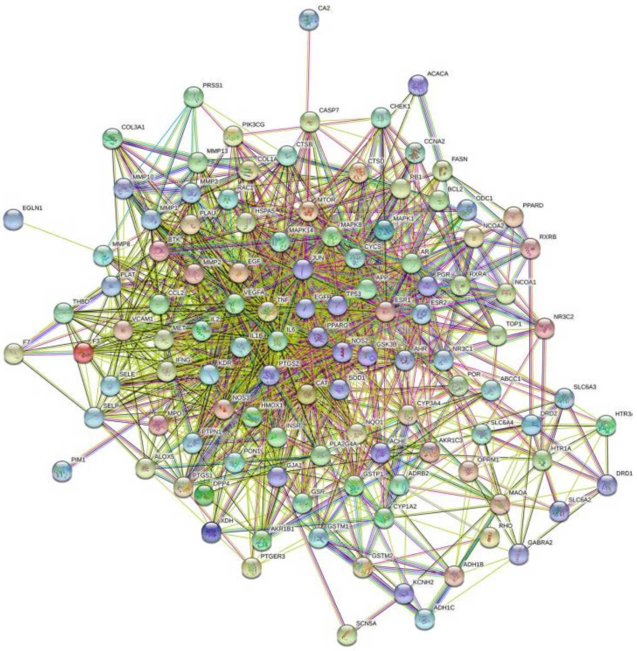

Supplementary Figure S2. **PPI network of SYF against BC**

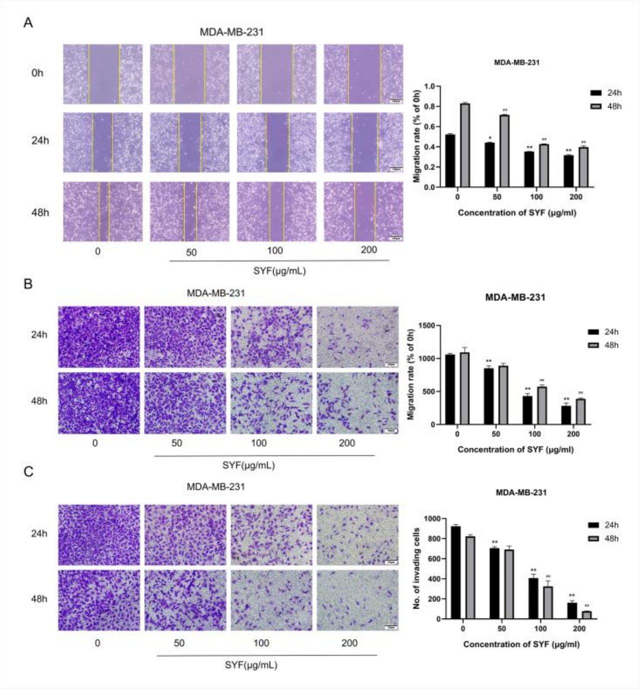

Supplementary Figure S3. **Effect of SYF extract on migration and invasion of MDA-MB-231 breast cancer cells** (A,B) Migration of MDA-MB-231 cells with SYF treatment was analyzed by wound healing assay (100×) and transwell assay (200×). (C) Invasion of MDA-MB-231 cells with SYF treatment was analyzed by invasion assay using Matrigel-coated transwell inserts (200×). *P<0.05, **P<0.01 vs the control group for 24 h; $^{\#}P$<0.05, $^{\#\#}P$<0.01 vs the control group for 48 h.

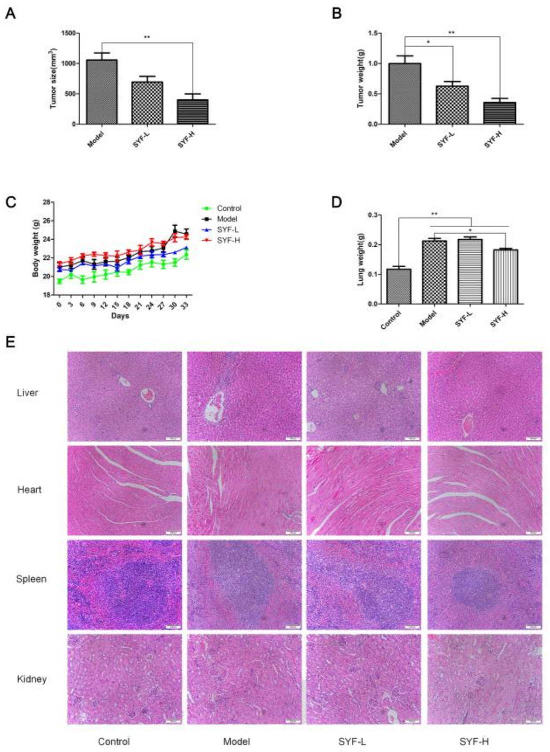

Supplementary Figure S4. **SYF inhibits the growth and lung metastasis of TNBC tumors, while also assessing the safety of the medication** (A,B) The weight and size of tumor was measured. (C) Body weight of mice during the experiment. (D) Lung weight of mice in different treatment groups. (E) H&E staining images of main organs with different doses of SYF extract (20×).
